# Hospital Building

**Published:** 1911-06-10

**Authors:** 


### HOSPITAL ARCHITECTURE AND CONSTRUCTION.

[Communications on this subject should be marked "Architecture" in the left-hand top corner of the envelope.]

### HOSPITAL BUILDING.

It is desirable and even necessary in handling a subject like Building of Hospitals that we should at the outset be clear as to what the terms we employ connote. Mr. A. Saxon Snell, F.R.I.B.A., in an interesting paper on the subject, read before the Society of Architects* gives as his first proposition that a hospital should be designed first and last for the housing and cure of the sick, diseased and injured, which is often overlooked or not realised in the design of the buildings, and further that good ventilation, heating and water supplies are consequential factors.

The designing of hospitals is a science which never reaches the zenith, for the conditions are ever changing, and each decade, it might even be said each year, brings developments which would make the conscientious architect wish to begin his well-considered problems over again in the newer aspect.

Mr. Snell gives a healthy reminder to those engaged in hospital planning by his reference to Miss Nightingale's "Treatise on Hospitals" published as far back as 1863, and emphasises that we have not even yet put into practice all the theories evolved and taught us by her long and close acquaintance with the realities of sickness and disease. An interesting allusion is made to the relative value of direct and diffused light in regard to health, a subject which when thoroughly investigated will probably revolutionise the present ideas of ward orientation.

Mr. Snell claims that the mortuary should be something more than merely and severely sanitary.

> "How wonderful is death,
> Death and its brother Sleep,
> . . . . So passing strange and wonderful,"

sang Shelley; and even the meanest of us will always feel awed in its presence; always by instinct pay respect to the cold clay. It seems to those full in Life a beautiful idea to make the mortuary a consecrated place.

* *Transactions of Society of Architects.* Price 1s.

